# Boundary cells regulate a switch in the expression of FGF3 in hindbrain rhombomeres

**DOI:** 10.1186/1471-213X-9-16

**Published:** 2009-02-20

**Authors:** Dalit Sela-Donenfeld, Galya Kayam, David G Wilkinson

**Affiliations:** 1 Koret School of Veterinary Medicine, The Hebrew University, The Robert H Smith Faculty of Agriculture, Food and Environment, PO Box 12, Rehovot 76100, Israel; 2 Division of Developmental Neurobiology, National Institute for Medical Research, The Ridgeway, Mill Hill, London, NW7 1AA, UK

## Abstract

**Background:**

During formation of the vertebrate central nervous system, the hindbrain is organized into segmental units, called rhombomeres (r). These cell-lineage restricted segments are separated by a subpopulation of cells known as boundary cells. Boundary cells display distinct molecular and cellular properties such as an elongated shape, enriched extracellular matrix components and a reduced proliferation rate compared to intra-rhombomeric cells. However, little is known regarding their functions and the mechanisms that regulate their formation.

**Results:**

Hindbrain boundary cells express several signaling molecules, such as FGF3, which at earlier developmental stages is transiently expressed in specific rhombomeres. We show that chick embryos that lack boundary cells due to overexpression of truncated EphA4 receptor in the hindbrain have continued segmental expression of *FGF3 *at stages when it is normally restricted to hindbrain boundaries. Furthermore, surgical ablation of the boundary between r3 and r4, or blocking of the contact of r4 with boundary cells, results in sustained *FGF3 *expression in this segment.

**Conclusion:**

These findings suggest that boundary cells are required for the downregulation of segmental *FGF3*, presumably mediated by a soluble factor(s) that emanates from boundaries. We propose that this new function of boundary cells enables a switch in gene expression that may be required for stage-specific functions of FGF3 in the developing hindbrain.

## Background

During early stages of nervous system development, the hindbrain is subdivided into several segments, called rhombomeres (r). Individual rhombomeres are polyclonal compartments, defined both by cell-lineage-restriction and by segmental expression of transcription factors, such as *Krox20*, *Kreisler *and members of the *Hox *gene family. This network of genes regulates the formation of specific rhombomeres and their identities along the anterior-posterior (A-P) axis [1-4]. The morphological and molecular segmentation of the hindbrain is essential for the establishment of specific patterns of neuronal differentiation and axon outgrowth and for the formation of distinct streams of migratory neural crest cells, implicated in the subsequent generation of neuronal networks and craniofacial structures (reviewed in [1-9]).

Concurrent with the establishment of hindbrain rhombomeres, a specialized population of boundary cells forms at their interfaces. A series of studies have characterized these cells, demonstrating that boundary cells have an elongated shape, increased extracellular spaces containing matrix components and that they display a reduced proliferation rate and interkinetic nuclear migration compared to intra-rhombomeric cells [10-12]. Much less is known regarding the mechanisms that regulate their formation or what the functions of boundary cells are during hindbrain development.

The signaling system of Eph tyrosine kinase receptors and their membrane-bound ephrin ligands have been shown to be required for boundary cell formation in zebrafish and *Xenopus *embryos [13,14]. Eph receptors and ephrins are largely expressed in alternate rhombomeres such that they interact at their interfaces, and this restricts cells from mixing across hindbrain segments, possibly by mediating cell repulsion [15-17]. In addition, EphA4 was shown to sharpen hindbrain boundaries by regulating cell affinity within rhombomeres [18]. Importantly, knocking down Eph/ephrin proteins or inhibition of their activation also leads to a decrease or loss of the expression of several boundary cell markers, such as *pax6 *and *sema3Gb*, in the zebrafish hindbrain [17,18]. These results indicate a requirement for this signaling system in boundary cell formation, although it is not known if this is due to a direct role in cell specification or secondary to the increased mixing between segments. Whether Eph-ephrin interactions are also required for hindbrain boundary formation in higher vertebrates is not known.

Several soluble signals were shown to be localized to boundary cells of different species. For instance,*Wnt1 *and *Wnt3a *are expressed in zebrafish hindbrain boundaries [19-21], while fibroblast growth factor 3 (*FGF3*) and *FGF19 *are confined to mouse and chick hindbrain boundary cells, from around E10/Hamburger Hamilton stage 16, respectively [22-26]. Some modulators or inhibitors of signaling systems, such as the TGFβ inhibitor follistatin and the Notch modulator radical fringe also accumulate at hindbrain boundaries of chick, mouse or zebrafish embryos [26-30]. Little is known regarding the function of these factors at rhombomere interfaces. Interestingly, before boundary cells are formed, several of these signals, such as *FGF3 *and *follistatin*, have segmental expression within specific rhombomeres [23,24,26-29]. The significance of these dynamic spatio-temporal expression patterns as well as the regulatory mechanisms by which these signals are turned on and off in different hindbrain regions are not clear.

In this study we set out to determine whether signaling by EphA4 is required for boundary cell formation in the chick hindbrain. We found that boundary cell markers and the formation of sharp interfaces were lost upon overexpression of dominant negative EphA4. Unexpectedly, we found that the segmental expression of the boundary cell marker *FGF3 *persisted, raising the possibility that lack of boundary cells may underlie the failure of rhombomeric *FGF3 *to become downregulated. The effect of ablation of boundary cells or blockage of the contact between a rhombomere and one of its boundaries confirmed this possibility. These findings suggest a new role for hindbrain boundary cells in inducing downregulation of the segmental expression of *FGF3 *in rhombomeres.

## Methods

### Eggs

Fertile Loman chick eggs were incubated at 38°C until embryos reached the desired somite-stage (ss). Before performing experimental procedures, eggs were windowed and embryos were visualised by injecting black ink below the blastodisc. Following manipulations, embryos were incubated to the required stage, fixed in 4% paraformaldehyde (PFA) and stored at -20°C for further analysis.

### *In ovo *electroporation

pCAGGS-IRES-GFP (a gift from J. Briscoe) and pCAGGS-truncated EphA4-IRES-GFP [17] constructs were diluted in 10 mM Tris pH 8.5 to a working concentration of 2 μg/μl. Vectors were injected into the hindbrain lumen of different staged embryos by using a pulled glass capillary. Following injection, electrodes were placed at the right and left sides of the embryo at hindbrain levels to obtain unilateral transfection. Electroporation was performed using a BTX 3000 electroporator with four 45 millisecond pulses of 12–16 volts and pulse intervals of 300 milliseconds [31].

### Whole-mount *in situ *hybridization and immunohistochemistry

Whole-mount *in situ *hybridization was performed as described [32], using chick probes for *hoxb1*, *FGF3 *(EST clone 812g6, MRC Geneservice, UK), *pax6 *(a gift from J. Briscoe), *Krox20 *(a gift from P. Charnay), *follistatin *(Connoly et al., 1995), and *NSCL1 *(EST clone 474F24, MRC Geneservice, UK). Probes were labeled with digoxigenin (DIG)-UTP and detected using alkaline phosphatase-coupled antibody (1:2000, Roche, Basel, Switzerland) followed by NBT/BCIP (Roche, Basel, Switzerland) staining. Whole-mount and paraffin-section immunohistochemistry was carried out alone or following some *in situ *hybridizations. Briefly, embryos were incubated in PBS with 0.1% Tween20, 5% goat serum for 2 hours (hrs) prior to incubation for 16 hrs with the following antibodies: rabbit anti-GFP (1:400, Molecular Probes, CA USA), rabbit anti-EphA4 (1:250) [33], mouse anti-chondroitin sulphate proteoglycan (CSPG, 1:50, Sigma, MO USA), as well as recombinant human ephrin A5-Fc (5 μg/ml, R&D systems, MN USA). Following PBS washes, the following secondary antibodies were added: anti-rabbit or anti-mouse Alexa 488 and anti-rabbit Alexa 594 (all 1:400, Molecular Probes, CA USA) to be visualized under epi-fluorescent microscope, or anti-rabbit and anti-mouse-HRP (1:250, Sigma, MO USA) visualized with AEC substrate system (Lab Vision Corporation, CA USA).

### *In ovo *microsurgery

For ablation of boundary cells, a rectangular cut was made with a pulled glass needle around the r3/r4 boundary region of 12–14 ss embryos and the tissue removed by aspiration. A silicon piece was cut to the precise size and inserted into the gap to prevent boundary regeneration. For barrier insertion, a transverse slit was made just posterior to r3/4 boundary or anterior to r4/5 boundary with a similar needle and the barrier was inserted into the slit. Barriers included either aluminum foil or a PCF membrane of 3 μm pores (Millipore, MA USA), both cut into adequate sizes. 1–2 days later, embryos were fixed in 4% PFA and prepared for *in situ *hybridization.

## Results

### Expression of truncated EphA4 receptor disrupts hindbrain segment borders

**Figure 1:**
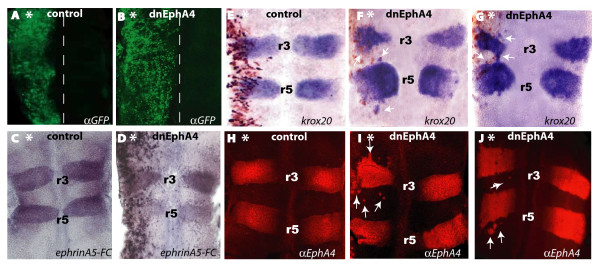
**Effects of truncated EphA4 on rhombomeres**. Flat-mounted dorsal views of chick embryonic hindbrains electroporated unilaterally with dnEphA4-IRES-GFP (B, D, F, G, I, J) or control pCAGGS-GFP (A, C, E, H,) constructs at 6–8 ss, then left to develop for further 24–40 hrs. (A, B): Hindbrains stained with anti-GFP antibody (midline is marked by dashed outlines). (C-D): Binding of ephrinA5-Fc chimera, which occurs in r3/r5, where endogenous Eph receptors are expressed (C, D) and also in ectopic domains corresponding to dnEphA4 electroporated cells (D). (E-G): *In situ *hybridized embryos using *Krox20 *probe show normal *Krox20 *expression in r3/r5 in control hindbrain (E) and ectopic *Krox20 *expression within r2/r4/r6 territories in dnEphA4-transfected hindbrains (F, G white arrows). (H-J): Confocal imaging of hindbrains stained with anti-EphA4 antibody showing cells expressing endogenous EphA4 within r2/r4/r6 domains on the dnEphA4-expressing side of the hindbrain (I-J, white arrows), compared to controls where EphA4 is localized to r3/r5 (H). Asterisks mark the electroporated side, treatments are stated at the bottom, anterior is at the top and rhombomeres (r) are numbered.

Previous studies have shown that overexpression of a dominant negative truncated form of mouse EphA4 (dnEphA4) in zebrafish embryos disrupts the formation of sharp interfaces and of boundary cells in the hindbrain [17]. We set out to investigate whether EphA4 has a similar role in the chick hindbrain by taking a similar approach. We cloned truncated mouse EphA4 into pCAGGS plasmid upstream to an internal ribosome entry site (IRES) and a GFP coding sequence to visualize its expression in the transfected cells. First, we assessed the expression level of the construct by electroporating it into the hindbrain of 6–8 ss chick embryos. High levels of GFP were found a day later in the electroporated cells which corresponded to dnEphA4 expression (Fig. 1B), similar to the expression of control GFP vector (Fig. 1A). Next, the ability of the dnEphA4 expressing cells to bind ephrin ligands was assessed by performing an *in situ *binding assay of a soluble ephrinA5-Fc chimeric protein to the dnEphA4 expressing cells. Strong levels of ephrinA5-Fc binding were found in ectopic locations in the dnEphA4 electroporated side of the hindbrain (Fig. 1D), in addition to r3 and r5 where endogenous Eph receptors are expressed, as seen in control electroporated embryos (Fig. 1C). Together, these results verify that truncated EphA4 is efficiently expressed and capable of binding its ligands in the chick hindbrain.

**Figure 2:**
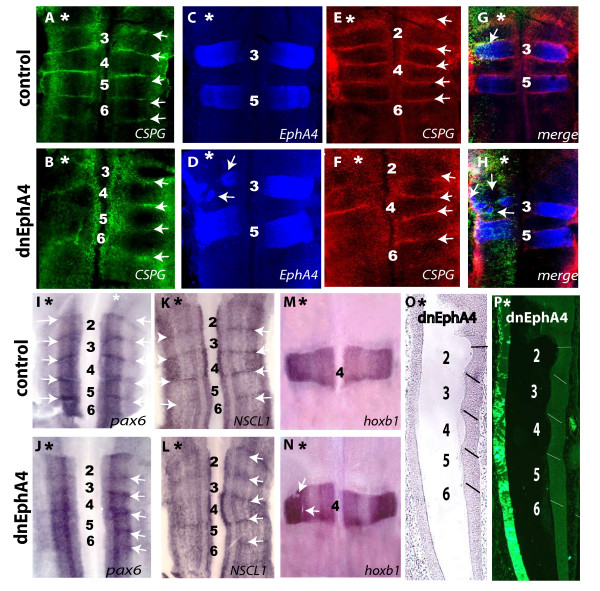
**Disruption of boundary cells in embryos expressing truncated EphA4**. Flat-mounted views of hindbrains electroporated unilaterally with dnEphA4- (B, D, F, H, J, L, N, O, P) or control (A, C, E, G, I, K, M) constructs at 6–8 ss, then left to develop for further 40 hrs. (A-H): Confocal imaging of hindbrains labeled with anti-CSPG or anti EphA4 antibodies, showing loss or disruption of boundary cells (B, F) and altered rhombomere shape (D, white arrows) upon dnEphA4-electroporation, compared to controls (A, C, E). (G, H): Overlay images of embryos shown in C-F, including GFP expression in the electroporated cells (green). (I-N): *In situ *hybridized embryos transfected with control (I, K, M) or dnEphA4 (J, L, N) constructs, using probes against *pax6 *(I, J), *NSCL1 *(K, L), *hoxb1 *(M, N). Unilateral loss or decrease in *pax6 *and *NSCL1 *boundary staining is observed in the dnEphA4-expressing hindbrains (J, L), in contrast to controls (I, K, white arrows). (M, N): *Hoxb1 *expression in r4 is altered or absent in some cells in the dnEphA4-trasfected side of the hindbrain (N, white arrows), in contrast to control (M). (O, P): Bright-field and fluorescent images of a frontal paraffin sectioned-embryo electroporated with dnEphA4. Typical morphology of a segmented hindbrain is evident in the control hemi-neural tube (boundaries are marked by lines), in contrast to the transfected side where constrictions and bulges are lost. Asterisks mark the electroporated side, labeling is stated at the bottom, anterior is at the top and rhombomeres are numbered.

To determine whether interfering with EphA4 signaling disrupts segmental gene expression, we examined the expression of the transcription factor *Krox20 *and its direct transcriptional target EphA4 [34], both normally sharply restricted to r3 and r5. In control electroporations, there was an expected r3 and r5 expression of *Krox20 *mRNA (Fig. 1E n = 12) and of EphA4 protein, which was detected with an antibody against the intracellular domain that binds the endogenous EphA4 but not the truncated ectopic protein (Fig. 1H n = 12). In contrast, there was a disruption to the formation of sharp borders on the electroporated side of embryos transfected with dnEphA4, with *Krox20 *and EphA4 expressing cells extending from their normal r3/r5 expression domains in the electroporated side into the adjacent r2, r4 or r6 territories (Fig. 1F, G, I, J and Fig. 2D; n = 12 for Krox20, n = 12 for EphA4). Notably, the data shows variability between embryos in the severity of the effects and in which segment(s) ectopic cells with *Krox20 *or EphA4 expression are observed in. This variability may be due to differences in electroporation efficiencies, intrinsic variability in cell mixing, and whether or not ectopic cells have switched their identity at the time of analysis [17]. Together, these results suggest that EphA4 signaling is required to restrict cell mixing between r3/r5 and adjacent segments in the chick hindbrain, consistent with previous reports in zebrafish and *Xenopus *embryos [17].

### Disruption of boundary cells in embryos expressing truncated EphA4

The disruption in sharp segmental borders raised the possibility that the formation of boundary cells at rhombomere interfaces is disturbed upon expression of truncated EphA4 receptor. To examine this, we analyzed the expression of several markers of chick hindbrain boundary cells. The extracellular matrix protein, chondroitin-sulphate proteoglycan (CSPG), accumulates at high levels at rhombomere boundaries [10] in control electroporations (Fig. 2A n = 10) and in the control side of dnEphA4-electroporated hindbrains (Fig. 2B, F right hand side). In contrast, accumulation of CSPG at boundaries is largely disrupted upon expression of dnEphA4 (Fig. 2B n = 10). Double staining with EphA4 and CSPG antibodies in another set of electroporated embryos confirmed that dnEphA4 transfection leads to disorganisation or loss of rhombomere boundaries together with distortion in the shape of rhombomeres on the electroporated side (Fig. 2D, F, H n = 6), compared to the non-electroporated side (Fig. 2D, F, H right hand side) and control embryos (Fig. 2C, E, G n = 6). Notably, in several embryos, r4 and its flanking boundaries are less affected by truncated EphA4 than other boundaries (Fig. 2B, F and also see Fig. 1E–J). A similar differential effect occurs following EphA4 knockdown in zebrafish embryos [18], which can be explained by the presence of Eph receptors in addition to EphA4 in r3/r5 that are activated by ephrins in r4, such that r4 boundaries are more resistant to decreased EphA4 function than other boundaries; further analysis will be required to determine whether a similar explanation holds in chick.

We next analysed the effect of dnEphA4 on the distribution of other boundary cell markers, the paired box gene *pax6 *[10] and the bHLH transcription factor *neural stem cell leukaemia 1 *(*NCSL1*) [35]. Both markers are expressed in a specific DV-restricted pattern along the hindbrain as well as in higher levels at rhombomere interfaces. We found that there is normal expression of both genes in control embryos (Fig. 2I, J n = 8 for each) and the non-electroporated side of embryos transfected with dnEphA4 (Fig. 2K, L right hand side n = 9 for each). In contrast, the elevated expression levels of *pax6 *(Fig. 2K n = 9) and of *NSCL1 *(Fig. 2L n = 9) at rhombomere borders decrease following overexpression of dnEphA4, whereas their DV-restricted expression within hindbrain segments appears unaffected. These findings suggest that disruption of Eph-ephrin signaling does not lead to a general alteration of gene expression throughout the hindbrain but rather to a localized effect at rhombomere interfaces. To further confirm this point, we evaluated the effect of dnEphA4 on the expression pattern *of hoxb1*, which is normally localized to r4. We found that in contrast to the sharp borders of r4 in controls (Fig. 2M n = 6), expression of dnEphA4 leads to a mild disruption in the formation of sharp domains of *hoxb1 *expression (Fig. 2N n = 6). Notably, as for Krox20 and EphA4, there was a disorganisation consistent with abnormal mixing rather than segmental changes to *hoxb1 *expression, suggesting that expression of dnEphA4 does not alter segmental identity.

Finally, the gross morphology of the manipulated hindbrain was examined by frontal paraffin sections of dnEphA4-expressing embryos. While the shape of rhombomere bulges separated by repeated boundary constrictions is evident in the intact side of the hindbrain (Fig. 2O, P right hand-side), a loss of this typical hindbrain morphology occurs on the dnEphA4 electroporated side (Fig. 2O, P left hand-side n = 5). Taken together, these results suggest that disruption of EphA4 signaling in the chick hindbrain leads to a decrease or loss of hindbrain boundary cells, in agreement with previous studies on zebrafish [17,18].

### The boundary cell marker *FGF3 *shows persistent segmental expression in embryos transfected with truncated EphA4 receptor

**Figure 3:**
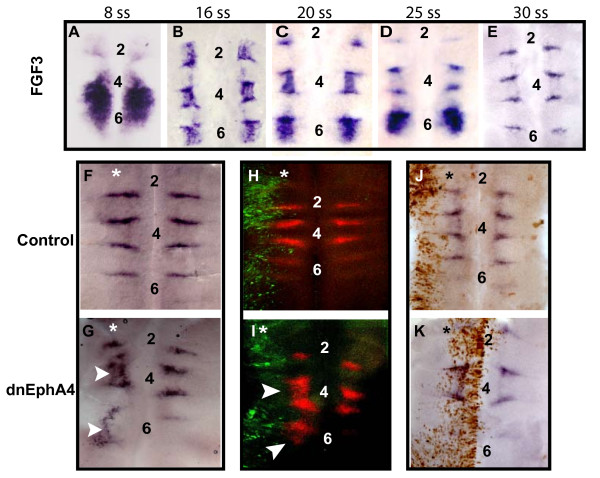
**The boundary cell marker *FGF3 *shows persistent segmental expression in embryos expressing truncated EphA4**. (A-E): Flat-mounted hindbrains from different-staged embryos *in situ *hybridized with *FGF3 *probe. (A): 8 ss embryo showing *FGF3 *expression in r4-r6 and low levels in r2. (B): 16 ss embryo showing expression in r2/r4/r6. (C): 20 ss embryos showing expression in r4/r6. *FGF3 *expression is also becoming apparent at rhombomere borders. (D): 25 ss embryo showing *FGF3 *transcripts in r6 and in boundaries. (E): 30 ss embryo showing *FGF3 *localization to boundary cells. (F-I): Flat-mounted hindbrains from embryos electroporated unilaterally with dnEphA4 (G, I) or control (F, H) constructs at 8 ss, and left to develop for further 30 hrs. *FGF3 *transcripts are restricted to boundaries in control electroporations (F, H), while dnEphA4-embryos show *FGF3 *within even-numbered rhombomeres (G, I white arrowheads). (J, K): Views of hindbrains electroporated at 22 ss with dnEphA4 (K) or control (J) constructs and left to develop for further 18 hrs. For both, expression of *FGF3 *is restricted to boundary cells. Embryos in (H-K) were immunostained with anti-GFP antibody followed by Alexa-488 (H, I) or HRP (J, K) secondary antibodies to label electroporated cells. Asterisks mark the electroporated side, anterior is at the top and rhombomere numbers are indicated.

*FGF3 *expression was previously described as occurring at early stages in specific hindbrain segments and later in hindbrain boundaries in chick and mouse embryos [22-24,26]. We carried out a more detailed analysis of the transition of *FGF3 *expression from a segmental pattern to boundary cells. In 8 somite stage (ss) embryos, *FGF3 *is expressed in the ventral half of r4-r6 and r6 (excluding the floor plate) and at lower levels in r2 (Fig. 3A). By 16 ss, expression is seen in all even-numbered segments, is downregulated in r5, and some upregulation begins at rhombomere borders (Fig. 3B). In 20 ss embryos, expression in r2 is downregulated while still present in the other even-numbered segments, and *FGF3 *transcripts become more apparent at r2/r3, r3/r4, r4/r5 and r5/r6 boundaries (Fig. 3C). By 25 ss, segmental expression of *FGF3 *is no longer detected in r4 while still present in r6, and expression in boundary cells has become even more prominent (Fig. 3D). Finally, in embryos at 30–45 ss, *FGF3 *transcripts are confined to the ventral part of hindbrain boundary cells and are absent from all rhombomere bodies (Fig. 3E). This analysis shows that *FGF3 *expression is firstly restricted to specific hindbrain segments and subsequently downregulated from these rhombomeres while expression is upregulated at rhombomere boundaries.

Our previous results show a loss in the expression of several boundary cell markers upon expression of a truncated form of EphA4 (Fig. 2). However, examination of *FGF3 *expression in 28–36 ss embryos following transfection of dnEphA4 at 8 ss revealed a surprising result:*FGF3 *was expressed in even-numbered rhombomeres (Fig. 3G, I, n = 12) at stages when this segmental expression would normally be downregulated (Fig. 3A–D). Indeed, on the contra-lateral side of the dnEphA4-expressing hindbrains (Fig. 3G, I right hand side) as well as in control electroporated embryos (Fig. 3F, H, n = 10), *FGF3 *expression was confined to boundary cells, as expected at this embryonic stage. The effect of dnEphA4 misexpression was non-cell autonomous since following dorsal electroporation, the transfected cells were not overlapping with the more ventral segmental *FGF3 *expression domains (Fig. 3I, compare GFP labeled cells in green with *FGF3 *expression in red). This result rules out the possibility that *FGF3 *becomes upregulated in cells expressing dnEphA4. In contrast to these findings, transfection of dnEphA4 in older embryos at 22 ss, when *FGF3 *is about to become downregulated from r4 (Fig. 3D), did not result in any rhombomeric expression of *FGF3*. Instead, expression occurred only in the boundary cells in both GFP control and dnEphA4 expressing embryos (Fig. 3J, K, n = 7 for each), further eliminating the possibility that ectopic expression of truncated Eph directly induces *FGF3 *upregulation. Taken together, these findings suggest that overexpression of dnEphA4 at stages when *FGF3 *is segmentally expressed leads to failure of the normal downregulation of this aspect of *FGF3 *expression.

### Downregulation of rhombomeric *FGF3 *requires boundary cells

**Figure 4:**
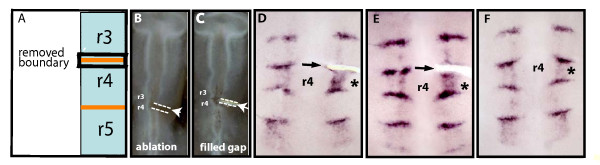
**Ablation of a boundary results in sustained rhombomeric expression of *FGF3***. (A-C): Diagram and photographs of a unilateral ablation of the r3/r4 boundary in 12 ss embryos (A, B), and after insertion of a silicon piece into the gap (C). (D-F): Flat-mounted hindbrains of embryos *in situ *hybridized with *FGF3 *probe at 28 ss. *FGF3 *continues to be expressed in r4 in the manipulated side (D, E, asterisks), while downregulated from r4 of the control side. In an embryo in which the ablated r3/r4 boundary was allowed to regenerate, *FGF3 *was downregulated from r4 and upregulated in the re-formed boundary (F, asterisk). In all images, anterior is at the top, arrows mark the position of the grafted piece and r4 is marked.

Potential clues to why dnEphA4 affects segmental *FGF3 *expression are the observations that this effect is non cell-autonomous (Fig. 3H, I) and that hindbrain boundary formation is disrupted upon blocking Eph-ephrin signaling (Fig. 2). A possible mechanism is that the presence of boundary cells is involved in the downregulation of *FGF3 *from hindbrain segments. Consistent with this, examination of the normal dynamics of *FGF3 *expression (Fig. 3A–E) shows the disappearance of *FGF3 *from even-numbered rhombomeres occurring later than the appearance of boundaries between hindbrain segments. Therefore, we took a microsurgical approach in order to examine whether rhombomeric *FGF3 *downregulation is affected in a segment where its adjacent boundary has been ablated. Unilateral removal of the r3/4 boundary was performed in 12 ss embryos, when this boundary is morphologically visible (Fig. 4A, B). As removal of a rhombomere boundary has been previously shown to be followed by its regeneration [36], we inserted a silicon piece into the ablated region to prevent interactions between r3 and r4 required for regeneration of the boundary (Fig. 4C). As a control for the microsurgery manipulation, some ablated embryos were allowed to develop without this insert to enable regeneration of the r3/4 boundary. Both types of embryos were fixed at 28 ss, when *FGF3 *is normally absent from r4 (Fig. 3D, E). Embryos that were lacking the r3/4 boundary were found to express *FGF3 *within r4 (Fig. 4D, E, n = 12), while on the contra-lateral side it was already downregulated from r4 and localized to the r3/4 and r4/5 boundaries. Moreover, *FGF3 *expression was already downregulated from r6 at both sides of this hindbrain (Fig. 4D, E), as expected from the embryonic stage at the time of fixation (Fig. 3D), indicating that the continued expression of *FGF3 *in r4 on the ablated side is not due to a general developmental delay. The ablation of the r3/4 boundary did not cause *FGF3 *expression in r3, a segment in which *FGF3 *is not expressed at any stage (Fig. 3A–E), indicating that the loss of r3/4 boundary does not result in an ectopic upregulation of *FGF3 *in the operated area. In contrast, in embryos in which the ablated r3/4 boundary was allowed to regenerate, *FGF3 *mRNA was not detected within r4 and was upregulated in the re-formed boundary as in normal embryos (Fig. 4F, n = 8). These data suggest that the r3/4 boundary is required in order for *FGF3 *to become downregulated from r4.

**Figure 5:**
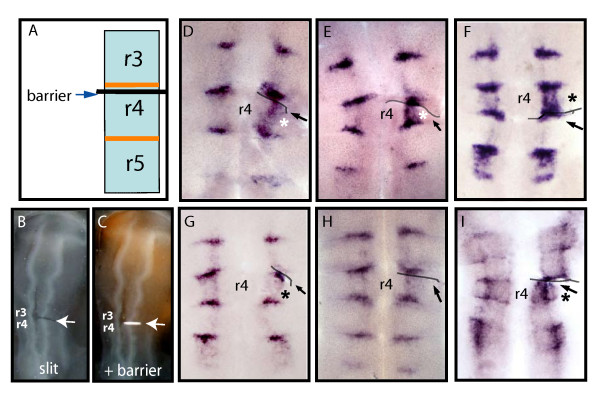
**Prevention of the contact between r4 and its boundaries results in sustained *FGF3 *expression in r4**. (A-C): Diagram and photographs of embryos with a unilateral slit between r4 and the r3/r4 boundary (B, arrow) and insertion of a barrier into the gap (A, C arrows) in 12ss embryos. (D-H): Flat-mounted hindbrains of *in situ *hybridized embryos stained for *FGF3 *at 28 ss. *FGF3 *remains expressed in r4 following insertion of a non-permeable barrier between r4 and r3/r4 boundary (D, E) or r4/r5 boundary (F) (asterisks), compared to the absence of *FGF3 *in r4 of the contra-lateral side. Barrier insertion between r3 and r3/4 boundary reveals no effect on *FGF3 *expression in either r3 or r4 (G, asterisk). Insertion of a permeable membrane between r3/r4 boundary and r4 results in *FGF3 *downregulation from r4 (H). (I): Flat-mounted hindbrain *in situ *hybridized with *follistatin *probe at 36 ss. *Follistatin *is expressed at higher levels in r4 following insertion of a non-permeable barrier between r4 and the r3/r4 boundary (I, asterisk), compared to the control side. In all images, anterior is at the top, grey lines and black arrows mark the barrier position, and r4 is marked.

Compartment boundaries act as organizing centers in several regions during vertebrate morphogenesis such as at the mid-hindbrain boundary and the limb bud. These centers regulate patterning and differentiation of the neighboring tissues by the production and secretion of soluble signals (reviewed in [37-39]). By analogy, our findings could be explained by the downregulation of *FGF3 *from hindbrain rhombomeres being mediated by soluble factor(s) from boundary cells. This possibility was examined by making a slit between the r3/4 boundary and r4 in 12 ss embryos, followed by insertion of a piece of aluminum foil as a non-permeable barrier into the slit (Fig. 5A–C). This manipulation aimed to block the secretion of a putative signal from this boundary towards r4 as well as to prevent cell processes, if present, from interacting between these two regions. Embryos were assessed for *FGF*3 expression at ~28 ss, a stage by which it is normally downregulated from rhombomeres (Fig. 3A–E). Embryos with the inserted barrier beneath r3/4 boundary had *FGF3 *expression within r4, while it has already been downregulated in this segment on the contra-lateral side (Fig. 5D, E n = 10). Notably, *FGF3 *expression was already downregulated from r6 on both sides of the manipulated hindbrain, arguing against the possibility that the microsurgery caused *FGF3 *to remain expressed in r4 due to a general delay in embryonic development. We next analyzed whether the effect on *FGF3 *in r4 is mediated only by the r3/r4 boundary or whether prevention of the contact between r4 and the r4/5 boundary will also prevent *FGF3 *from becoming downregulated from r4. We found that insertion of a barrier between r4 and the r4/5 boundary led to persistent segmental expression of *FGF3 *(Fig. 5F n = 6). This result shows that both boundaries contribute to the downregulation of *FGF3 *from r4, and suggests that the concentration of the putative factor(s) that is released from both boundaries is limiting such that reduction in its amount from either boundary is sufficient to prevent *FGF3 *from being downregulated.

The finding that disruption of boundaries by expression of dnEphA4 leads to sustained segmental expression of *FGF3 *(Fig. 3F–I) argues against the alternative explanation that the effects of barrier insertion are due to disruption of signaling between segments rather than the absence of a normal border in-between. Nevertheless, to test this we analyzed whether inserting a barrier anterior to the r3/4 boundary affects *FGF3 *expression. We found that this did not result in sustained *FGF3 *expression in r4 or in ectopic expression in r3 (Fig. 5G, n = 5). This result further confirms that *FGF3 *expression in r4 (Fig. 5D, E) is not due to a non-specific effect of the operation procedure. Moreover, this indicates that it is not signaling from r3 to r4 that is necessary to downregulate *FGF3 *in r4, but rather the r3/4 boundary itself is required.

To further analyze whether signaling from boundaries in involved in downregulation of segmental *FGF3 *expression, we inserted a permeable membrane of 3 μm-diameter pore size, which allows diffusion of proteins, between the r3/4 boundary and r4. In these embryos, *FGF3 *was downregulated from this segment and localized to the boundaries, as in the contra-lateral side of the operated embryo (Fig. 5H, n = 11). This control further excludes the possibility that the sustained *FGF3 *expression in r4 observed upon the insertion of the non-permeable barrier (Fig. 5D–F) is a non-specific effect of the surgical manipulation. The differing effects of inserting a non-permeable (Fig. 5D–F) or a porous barrier (Fig. 5H) suggests that a diffusible factor(s) from r4 boundaries are involved in downregulation of *FGF3 *from this segment.

Finally, we analyzed whether boundary cells also regulate the expression of *follistatin*, which is initially expressed in a segmental pattern in several rhombomeres including r4, and later becomes restricted to boundary cells and a DV-restricted stripe [26]. We found that prevention of the contact between r4 and its anterior boundary at 12 ss embryos resulted in higher levels of *follistain *expression in r4 in 35 ss embryos (Fig. 5I n = 8). Due to the normal DV-restricted expression of *follistatin*, this result is less clear-cut than for *FGF3*, but nevertheless argues that boundaries regulate the expression of multiple genes within r4.

## Discussion

*FGF3 *has dynamic expression in the chick embryo hindbrain in which it is first restricted to specific rhombomeres and later becomes downregulated from these segments and upregulated at rhombomere boundaries. This study shows that embryos that lack hindbrain boundary cells, due to either surgical ablation or overexpression of a truncated EphA4 receptor, maintain segmental expression of *FGF3 *at stages when it is normally restricted to hindbrain boundaries. Moreover, abnormal maintenance of *FGF3 *expression in r4 occurs upon insertion of a non-permeable barrier between this rhombomere and either of its boundaries, suggesting that the downregularion of segmental *FGF3 *is induced due to the secretion of signals from boundary cells. These results suggest a novel function of boundary cells in promoting the downregulation of segmental expression of *FGF3 *in hindbrain segments.

### Eph receptors are required for boundary formation in the chick hindbrain

The inhibition of cell mixing between adjacent rhombomeres is crucial to establish sharp domains of segmental gene expression in the hindbrain. Signaling by Eph receptors and their ephrin ligands have a key role in the sharpening of boundaries in the zebrafish hindbrain, by interactions across boundaries that restrict cell mixing between segments as well as by regulating cell affinity within rhombomeres [17,18]. Our results in the chick embryo, in which expression of dnEphA4 leads to mixing of cells with r3/r5 identities into even numbered territories, suggest that Eph-ephrin signaling is required for hindbrain boundary sharpening in a higher vertebrate.

Analysis of multiple markers of hindbrain boundaries and of hindbrain morphology shows that formation of boundary cells is severely disrupted in the chick hindbrain following expression of truncated EphA4. These results are in agreement with previous reports in zebrafish where inhibition or knockdown of EphA4 function leads to loss of *pax6 *or *sema3Gb *expression in boundaries [17,18]. It is possible that Eph-ephrin signaling at rhombomere interfaces specifies boundary cells directly, or by inhibiting cell mixing, provides a stable interface between rhombomeres that is essential for boundary cells to form.

### Potential significance of switch in *FGF3 *gene expression

Our finding that signaling from hindbrain boundaries promotes the downregulation of the segmental expression of *FGF3 *raises the question of the potential role of this interaction. The dynamic regulation of *FGF3 *in the hindbrain may be an example of the widespread phenomenon of the redeployment of the same signals at sequential stages of development. *FGF3 *and several other secreted factors, such as *FGF19 *and *follistatin*, initially have rhombomeric expression that then switches to boundary-restricted expression in the chick and mouse embryo hindbrain [22-25,27-29,40]. The early segmental expression of *FGF3 *is required for the induction and patterning of the otic vesicle, which develops from a placode adjacent to the hindbrain [41-43]. In addition, rhombomeric-derived FGFs are required for hindbrain patterning, such as segmental expression of *Krox20 *and *Kreisle*r/MafB in specific segments [44-46]. Recently, we have found that the segmental expression of *FGF3 *is enabled by follistatin that is expressed in the same segments and blocks BMP signaling that would otherwise inhibit the upregulation of *FGF3 *[26]. Knockdown of follistatin leads to a lack of segmental *FGF3 *expression and disruption to hindbrain patterning [26].

The downregulation of segmental *FGF3 *expression could simply reflect that its initial roles in hindbrain segmentation and inner ear induction have been fulfilled, so continued expression is not required. However, our finding that boundary signals promote the downregulation of *FGF3 *suggests a more active requirement. An attractive possibility is that continued segmental expression of *FGF3 *would interfere with the roles of the subsequent boundary-restricted expression. Currently, it is not possible to test this since the role of *FGF3 *expression at hindbrain boundaries is not known. The expression of a number of signaling molecules at hindbrain boundaries in chick [22-29,40,47] is suggestive of roles as signaling centers that pattern adjacent rhombomeres. Such a role could be analogous to the midbrain-hindbrain boundary that acts as an organizing centre to pattern cell fate and neural differentiation in the midbrain and anterior hindbrain through the secretion of FGF8 [37,39,48-50]. However, it is not clear whether hindbrain boundaries organize neuronal patterns within rhombomeres in the chick since, unlike the situation in zebrafish hindbrain [51], there is no overt organization of repetitive neurogenesis or of mature neurons and glia along the AP axis within rhombomeres [1,52]. Furthermore, there is normal formation of neuronal nuclei following retinoic acid treatment, surgical ablation or genetic alterations that disrupt hindbrain boundaries in chick or mouse embryos [36,53-55]. Nevertheless, it is possible that FGFs expressed at hindbrain boundaries have roles in other aspects of tissue organization, such as axon pathfinding or formation of nerve exit points [36,52,53].

### What is the signal that downregulates FGF3?

An important question raised by our results is the identity of boundary signals that induce the downregulation of segmental *FGF3 *in the hindbrain. One possibility is that segmental – but not boundary – *FGF3 *expression is itself downregulated by FGF signaling from boundaries. However, on the contrary we found that addition of exogenous *FGF3 *increased the level of segmental *FGF3 *expression whereas blocking of FGF receptors inhibited *FGF3 *expression [26]. These results suggest that an autoregulatory positive feedback loop regulates *FGF3 *expression in the hindbrain, arguing against the possibility that *FGF3 *from boundaries downregulates its own expression in segment bodies. *FGF3 *downregulation may be mediated by the loss of *follistatin *from segment bodies, since both genes overlap in the hindbrain and we have recently found that *FGF3 *requires follistatin in order to be expressed in rhombomeres [26]. Our present data are consistent with the possibility that a boundary signal(s) downregulates segmental *follistatin *expression, in turn leading to downregulation of *FGF3*. Further investigations are required to elucidate which secreted boundary signals are inducing the downregulation of *FGF3 *and *follistatin *from rhombomeres.

## Conclusion

In conclusion, we demonstrate that interference with EphA4 signaling in the chick hindbrain prevents the formation of sharp rhombomere interfaces. In addition, boundary cell markers are lost upon overexpression of dominant negative EphA4. However, the segmental expression of the boundary cell marker *FGF3 *persists. Similar sustained expression of *FGF3 *in rhombomeres occurs upon ablation of boundary cells or blockage of the contact between a rhombomere and its boundaries. Together, we suggest that hindbrain boundary cells are required for the switching-off of rhombomeric *FGF3*, presumably mediated by a soluble factor(s) emanating from the boundaries. These findings imply for a new role of boundary cells in the downregulation of genes expressed at hindbrain segments. This boundary-cell activity may be required for stage-specific function of segmental genes in the developing hindbrain.

## Authors' contributions

DSD designed and guided the study, carried out most of the experiments, analyzed and interpreted the data, and wrote the manuscript. KG carried out several experiments and in situ hybridizations, collected data and helped drafting the manuscript. DGW guided the study, helped with interpretations and edited the manuscript. All authors read and approved the final manuscript.

## References

[B1] Lumsden A, Krumlauf R (1996). Patterning the vertebrate neuraxis. Science.

[B2] Lumsden A (2004). Segmentation and compartition in the early avian hindbrain. Mech Dev.

[B3] Moens CB, Prince VE (2002). Constructing the hindbrain: insights from the zebrafish. Dev Dyn.

[B4] Guthrie S (1996). Patterning the hindbrain. Curr Opin Neurobiol.

[B5] Trainor PA, Krumlauf R (2000). Patterning the cranial neural crest: hindbrain segmentation and Hox gene plasticity. Nat Rev Neurosci.

[B6] Schneider-Maunoury S, Gilardi-Hebenstreit P, Charnay P (1998). How to build a vertebrate hindbrain. Lessons from genetics. C R Acad Sci III.

[B7] Chatonnet F, Borday C, Wrobel L, Thoby-Brisson M, Fortin G, McLean H, Champagnat J (2006). Ontogeny of central rhythm generation in chicks and rodents. Respir Physiol Neurobiol.

[B8] Rijli FM, Gavalas A, Chambon P (1998). Segmentation and specification in the branchial region of the head: the role of the Hox selector genes. Int J Dev Biol.

[B9] Gavalas A, Krumlauf R (2000). Retinoid signalling and hindbrain patterning. Curr Opin Genet Dev.

[B10] Heyman I, Faissner A, Lumsden A (1995). Cell and matrix specialisations of rhombomere boundaries. Dev Dyn.

[B11] Heyman I, Kent A, Lumsden A (1993). Cellular morphology and extracellular space at rhombomere boundaries in the chick embryo hindbrain. Dev Dyn.

[B12] Guthrie S, Butcher M, Lumsden A (1991). Patterns of cell division and interkinetic nuclear migration in the chick embryo hindbrain. J Neurobiol.

[B13] Xu Q, Mellitzer G, Wilkinson DG (2000). Roles of Eph receptors and ephrins in segmental patterning. Philos Trans R Soc Lond B Biol Sci.

[B14] Cooke JE, Moens CB (2002). Boundary formation in the hindbrain: Eph only it were simple. Trends Neurosci.

[B15] Mellitzer G, Xu Q, Wilkinson DG (1999). Eph receptors and ephrins restrict cell intermingling and communication. Nature.

[B16] Xu Q, Mellitzer G, Robinson V, Wilkinson DG (1999). In vivo cell sorting in complementary segmental domains mediated by Eph receptors and ephrins. Nature.

[B17] Xu Q, Alldus G, Holder N, Wilkinson DG (1995). Expression of truncated Sek-1 receptor tyrosine kinase disrupts the segmental restriction of gene expression in the Xenopus and zebrafish hindbrain. Development.

[B18] Cooke JE, Kemp HA, Moens CB (2005). EphA4 is required for cell adhesion and rhombomere-boundary formation in the zebrafish. Curr Biol.

[B19] Riley BB, Chiang MY, Storch EM, Heck R, Buckles GR, Lekven AC (2004). Rhombomere boundaries are Wnt signaling centers that regulate metameric patterning in the zebrafish hindbrain. Dev Dyn.

[B20] Amoyel M, Cheng YC, Jiang YJ, Wilkinson DG (2005). Wnt1 regulates neurogenesis and mediates lateral inhibition of boundary cell specification in the zebrafish hindbrain. Development.

[B21] Dorsky RI, Itoh M, Moon RT, Chitnis A (2003). Two tcf3 genes cooperate to pattern the zebrafish brain. Development.

[B22] Wilkinson DG, Peters G, Dickson C, McMahon AP (1988). Expression of the FGF-related proto-oncogene int-2 during gastrulation and neurulation in the mouse. Embo J.

[B23] Mahmood R, Kiefer P, Guthrie S, Dickson C, Mason I (1995). Multiple roles for FGF-3 during cranial neural development in the chicken. Development.

[B24] Mahmood R, Mason IJ, Morriss-Kay GM (1996). Expression of Fgf-3 in relation to hindbrain segmentation, otic pit position and pharyngeal arch morphology in normal and retinoic acid-exposed mouse embryos. Anat Embryol (Berl).

[B25] Kurose H, Bito T, Adachi T, Shimizu M, Noji S, Ohuchi H (2004). Expression of Fibroblast growth factor 19 (Fgf19) during chicken embryogenesis and eye development, compared with Fgf15 expression in the mouse. Gene Expr Patterns.

[B26] Weisinger K, Wilkinson DG, Sela-Donenfeld D (2008). Inhibition of BMPs by follistatin is required for FGF3 expression and segmental patterning of the hindbrain. Dev Biol.

[B27] Albano RM, Arkell R, Beddington RS, Smith JC (1994). Expression of inhibin subunits and follistatin during postimplantation mouse development: decidual expression of activin and expression of follistatin in primitive streak, somites and hindbrain. Development.

[B28] Connolly DJ, Patel K, Seleiro EA, Wilkinson DG, Cooke J (1995). Cloning, sequencing, and expressional analysis of the chick homologue of follistatin. Dev Genet.

[B29] Graham A, Lumsden A (1996). Interactions between rhombomeres modulate Krox-20 and follistatin expression in the chick embryo hindbrain. Development.

[B30] Cheng YC, Amoyel M, Qiu X, Jiang YJ, Xu Q, Wilkinson DG (2004). Notch activation regulates the segregation and differentiation of rhombomere boundary cells in the zebrafish hindbrain. Dev Cell.

[B31] Itasaki N, Bel-Vialar S, Krumlauf R (1999). 'Shocking' developments in chick embryology: electroporation and in ovo gene expression. Nat Cell Biol.

[B32] Sela-Donenfeld D, Kalcheim C (1999). Regulation of the onset of neural crest migration by coordinated activity of BMP4 and Noggin in the dorsal neural tube. Development.

[B33] Irving C, Nieto MA, DasGupta R, Charnay P, Wilkinson DG (1996). Progressive spatial restriction of Sek-1 and Krox-20 gene expression during hindbrain segmentation. Dev Biol.

[B34] Theil T, Frain M, Gilardi-Hebenstreit P, Flenniken A, Charnay P, Wilkinson DG (1998). Segmental expression of the EphA4 (Sek-1) receptor tyrosine kinase in the hindbrain is under direct transcriptional control of Krox-20. Development.

[B35] Theodorakis K, Kyriakopoulou K, Wassef M, Karagogeos D (2002). Novel sites of expression of the bHLH gene NSCL1 in the developing nervous system. Mech Dev.

[B36] Guthrie S, Lumsden A (1991). Formation and regeneration of rhombomere boundaries in the developing chick hindbrain. Development.

[B37] Kiecker C, Lumsden A (2005). Compartments and their boundaries in vertebrate brain development. Nat Rev Neurosci.

[B38] Ng JK, Tamura K, Buscher D, Izpisua-Belmonte JC (1999). Molecular and cellular basis of pattern formation during vertebrate limb development. Curr Top Dev Biol.

[B39] Zervas M, Blaess S, Joyner AL (2005). Classical embryological studies and modern genetic analysis of midbrain and cerebellum development. Curr Top Dev Biol.

[B40] Powles N, Marshall H, Economou A, Chiang C, Murakami A, Dickson C, Krumlauf R, Maconochie M (2004). Regulatory analysis of the mouse Fgf3 gene: control of embryonic expression patterns and dependence upon sonic hedgehog (Shh) signalling. Dev Dyn.

[B41] Zelarayan LC, Vendrell V, Alvarez Y, Dominguez-Frutos E, Theil T, Alonso MT, Maconochie M, Schimmang T (2007). Differential requirements for FGF3, FGF8 and FGF10 during inner ear development. Dev Biol.

[B42] Wright TJ, Mansour SL (2003). Fgf3 and Fgf10 are required for mouse otic placode induction. Development.

[B43] Phillips BT, Bolding K, Riley BB (2001). Zebrafish fgf3 and fgf8 encode redundant functions required for otic placode induction. Dev Biol.

[B44] Marin F, Charnay P (2000). Hindbrain patterning: FGFs regulate Krox20 and mafB/kr expression in the otic/preotic region. Development.

[B45] Maves L, Jackman W, Kimmel CB (2002). FGF3 and FGF8 mediate a rhombomere 4 signaling activity in the zebrafish hindbrain. Development.

[B46] Walshe J, Maroon H, McGonnell IM, Dickson C, Mason I (2002). Establishment of hindbrain segmental identity requires signaling by FGF3 and FGF8. Curr Biol.

[B47] Walshe J, Mason I (2000). Expression of FGFR1, FGFR2 and FGFR3 during early neural development in the chick embryo. Mech Dev.

[B48] Raible F, Brand M (2004). Divide et Impera – the midbrain-hindbrain boundary and its organizer. Trends Neurosci.

[B49] Wurst W, Bally-Cuif L (2001). Neural plate patterning: upstream and downstream of the isthmic organizer. Nat Rev Neurosci.

[B50] Wassef M, Joyner AL (1997). Early mesencephalon/metencephalon patterning and development of the cerebellum. Perspect Dev Neurobiol.

[B51] Trevarrow B, Marks DL, Kimmel CB (1990). Organization of hindbrain segments in the zebrafish embryo. Neuron.

[B52] Lumsden A, Keynes R (1989). Segmental patterns of neuronal development in the chick hindbrain. Nature.

[B53] Nittenberg R, Patel K, Joshi Y, Krumlauf R, Wilkinson DG, Brickell PM, Tickle C, Clarke JD (1997). Cell movements, neuronal organisation and gene expression in hindbrains lacking morphological boundaries. Development.

[B54] Carpenter EM, Goddard JM, Chisaka O, Manley NR, Capecchi MR (1993). Loss of Hox-A1 (Hox-1.6) function results in the reorganization of the murine hindbrain. Development.

[B55] Baek JH, Hatakeyama J, Sakamoto S, Ohtsuka T, Kageyama R (2006). Persistent and high levels of Hes1 expression regulate boundary formation in the developing central nervous system. Development.

